# Automated analysis of facial emotions in subjects with cognitive impairment

**DOI:** 10.1371/journal.pone.0262527

**Published:** 2022-01-21

**Authors:** Zifan Jiang, Salman Seyedi, Rafi U. Haque, Alvince L. Pongos, Kayci L. Vickers, Cecelia M. Manzanares, James J. Lah, Allan I. Levey, Gari D. Clifford

**Affiliations:** 1 Department of Biomedical Informatics, Emory University School of Medicine, Atlanta, GA, United States of America; 2 Department of Biomedical Engineering, Georgia Institute of Technology and Emory University, Atlanta, GA, United States of America; 3 Department of Neurology, Emory University School of Medicine, Atlanta, GA, United States of America; Nathan S Kline Institute, UNITED STATES

## Abstract

Differences in expressing facial emotions are broadly observed in people with cognitive impairment. However, these differences have been difficult to objectively quantify and systematically evaluate among people with cognitive impairment across disease etiologies and severity. Therefore, a computer vision-based deep learning model for facial emotion recognition trained on 400.000 faces was utilized to analyze facial emotions expressed during a passive viewing memory test. In addition, this study was conducted on a large number of individuals (n = 493), including healthy controls and individuals with cognitive impairment due to diverse underlying etiologies and across different disease stages. Diagnoses included subjective cognitive impairment, Mild Cognitive Impairment (MCI) due to AD, MCI due to other etiologies, dementia due to Alzheimer’s diseases (AD), and dementia due to other etiologies (e.g., Vascular Dementia, Frontotemporal Dementia, Lewy Body Dementia, etc.). The Montreal Cognitive Assessment (MoCA) was used to evaluate cognitive performance across all participants. A participant with a score of less than or equal to 24 was considered cognitively impaired (CI). Compared to cognitively unimpaired (CU) participants, CI participants expressed significantly less positive emotions, more negative emotions, and higher facial expressiveness during the test. In addition, classification analysis revealed that facial emotions expressed during the test allowed effective differentiation of CI from CU participants, largely independent of sex, race, age, education level, mood, and eye movements (derived from an eye-tracking-based digital biomarker for cognitive impairment). No screening methods reliably differentiated the underlying etiology of the cognitive impairment. The findings provide quantitative and comprehensive evidence that the expression of facial emotions is significantly different in people with cognitive impairment, and suggests this may be a useful tool for passive screening of cognitive impairment.

## Introduction

Alzheimer’s disease (AD) is the most common form of dementia that typically presents with memory loss due to neurodegeneration of temporal lobe brain circuits and other regions involved in learning and memory [1]. Mild Cognitive Impairment due to AD (AD-MCI) is a prodromal stage in the AD continuum, where cognitive problems first become noticeable to family members and friends. This stage is therefore considered an early window for detecting cognitive impairment before the stark cognitive, behavioral and functional decline associated with progression to dementia. Neuropsychiatric symptoms also occur frequently early in the disease process [2], but these non-cognitive symptoms are often unrecognized.

In addition to measuring biochemical biomarkers like beta-amyloid in cerebrospinal fluid (CSF) or using positron emission tomography (PET) scans, assessing objective cognitive impairment with neuropsychological measures is an important clinical criterion for the diagnosis of MCI and dementia [1]. Included in these assessments are general cognitive screening tools such as the Montreal Cognitive Assessment (MoCA) [3] or Mini Mental Status Exam [4]. Although these tools have been shown utility for screening for MCI [5], it is challenging to scale these types of assessments, mainly because they need to be administered by trained personnel. In recent work we have studied passive activity of the face from webcams on consumer hardware in a psychiatric population [6]. Here we extend that work to study the nature of emotional expression in cognitively impaired subjects.

A broadly observed difference in people with cognitive impairment is difficulty recognizing and processing facial expressions [7–18], which may provide an early signal to family and friends for seeking a diagnosis or initiating treatment. Abnormalities in different brain regions, such as frontal regions and anterior cingulate, were also revealed in previous neuroimaging studies on emotion regulation in AD-D and MCI [19, 20]. Significant differences in facial emotion recognition were described and assessed in patients with AD dementia (AD-D) [7, 8] and MCI [7, 9, 10].

For the expression of facial emotions, most studies focused on patients with mid- to late-stage AD. Some studies investigated facial expressions of AD patients when viewing emotion-eliciting images or videos and found their subjective emotional experience of the elicited emotion to be preserved [11, 12], while the subjective experience of the non-target emotions (the emotions that were not designed to be elicited) was found to be increased [13]. In addition, their abilities in regulating facial expression were reported impaired. Seidl *et al*. [14] reported that cognitive decline was related to increased facial expressiveness (average frequency of emotion expression during viewing emotion-eliciting and neutral images) after controlling apathy in patients with AD. In addition, zygomatic activity was found to be different in patients with AD while viewing emotion-eliciting images, when compared to healthy elderly controls [11]. The flexibility of the emotion expression was also reported to be impaired in patients with AD. More specifically, they were less effective, compared to the controls, in amplifying their positive affect when they were requested to do so [12]. Other studies have investigated the facial expressions of patients with dementia during daily activities. For example, Lee *et al*. reported that the quantity of positive emotional expression was positively related to wandering rates [15]. Unlike the increased facial expressiveness reported in emotion-eliciting studies [13], a lower frequency of expressive behavior in dementia patients has been reported [16], while the functional relation to patient likes and dislikes was represented in the emotional expressions.

Patients with other neurodegenerative disorders also manifest abnormalities in producing facial expressions. For example, slack facial expression was more common in people with dementia with Lewy bodies (DLB) [17], and people with Parkinson’s disease with dementia (PD-D) also have the symptom of hypomimia [18].

Although many studies investigated the symptomatic change of facial expressions in people with cognitive impairment they each have their limitations. The first limitation is that most did not consider patients with MCI, which made it unclear how early abnormal facial expressions are present in disease course and precluded face processing as a marker for early detection. The second limitation is that none of the studies systematically compared the pattern of facial expressions across different stages of disease (e.g., MCI or dementia) or across underlying etiologies (such as AD and other neurodegenerative conditions), which would have provided a better understanding of each condition. For instance, facial expressiveness was reported to be decreased in people with DLB or PD-D [17, 18] but increased in people with AD-D [14]. The third limitation is that most of the studies either made subjective observations of facial expression, or experts were required to encode action units of the face and measure physiological signals correlated with emotions: the former is susceptible to low-inter-rater reliability, and the latter often fails to scale. Our approach sought to overcome these limitations. To overcome the challenges in previous facial expression assessment methods, we adapted a deep learning-based framework we previously developed for predicting remission from depression [6], which was trained on approximately 840,000 faces, and was shown to achieve acceptable accuracy in recognizing facial emotions.

In this work, we hypothesized that reference facial expressions (those expressed during a passive viewing task) would be different in people with cognitive impairment when compared to a healthy population. Furthermore, cognitively impaired subjects may exhibit different expressions or emotions at different stages, or for varying types of impairment. To test these hypotheses, we acquired videos of healthy controls (HCs) and participants with AD-D, AD-MCI, and varied types of non-AD dementia (non-AD-D) or non-AD MCI during a passive viewing memory test, which was not designed to elicit specific emotions. Images were of landscapes, art, and humans or animals in everyday situations.

## Materials and methods

### Participants

A total of 493 participants were recruited from the Emory Healthy Brain Study (EHBS, n = 258) and the Goizueta Alzheimer’s Disease Research Center-affiliated clinics (ADRC, n = 235) at Emory University. The ADRC participants (n = 235) consisted of participants at different stages of AD and other types of cognitive impairment. Research diagnoses for the ADRC group were determined by the ADRC consensus review committee consisting of neurologists, neuropsychologists, and psychometrists who assessed the participants. Categorization into diagnostic groups represented a clinical judgment based on combined results of medical history, clinical exam, and cognitive assessment. The severity of decline (e.g., MCI or dementia) was based upon cognitive and functional status, consistent with DSM-5 diagnoses. Participants were later categorized into specific subgroups relevant for this study (e.g., MCI-AD, MCI-Non-AD, Dementia-AD, Dementia-Non-AD) by clinical review of recent clinical notes from their neurologist and available cognitive testing. General comorbidities (e.g., high blood pressure) were not accounted for in the present analysis.

The demographics of the participants can be found in Table 1. All procedures followed were in accordance with the ethical standards of the responsible committee on human experimentation.

**Table 1:** Demographics of the 493 participants grouped by MoCA.

	MoCA<= 24 (CI)	MoCA>24 (CU)
Subject Number	256	237
Age (years)	73.3 ± 8.7	67.5 ± 8.7
Sex (M/F ratio)	55/62*	31/28*
Race (C/AA/Oth)	174/42/2*	100/14/10*
Years of Education	15.7 ± 2.5	16.9 ± 2.1
MoCA Score	17.6 ± 5.8	27.2 ± 1.6

Note: C = Caucasian; AA = African American; Oth = Other (including American Indian, Alaska native and Pacific Islander).

* *Data* are only available for a subset of participants. 151 (30.6%) among the 493 subjects do not have race information, and 314 (63.7%) among the 493 subjects do not have the sex information.

± indicates the standard deviation of the measured variable. The year of education indicates the number of academic years a person completed in a formal program provided by elementary and secondary schools, universities, colleges, or other formal post-secondary institutions. Completion of high school usually corresponds to 12 years of education, where completion of college usually corresponds to 16 years of education.

#### Capacity to provide consent

Special considerations are necessary for those adults with Alzheimer’s disease and related disorders that affect cognitive abilities and thus have the potential to impair an individual’s capacity to understand and provide consent. To address this concern, we ensured that the individual(s) signing the assent/consent form, whether the participant themselves or the participant’s representative, have a full understanding of the study. Those providing consent were asked to reiterate what they understand to be the primary goal of this study, the risks, benefits, and requirements of participation. Although some participants with dementia are competent, dual consent from the participant and their representative is obtained prior to enrollment in the study. The consent procedure and this study have been formally approved by the Emory University Institutional Review Board (IRB00078273).

### Measurements

All participants received evaluations that included neuropsychological testing and evaluation of mood. The MoCA (version 8.1) [3] was used as a common screening measure to evaluate global cognitive performance in both the EHBS and ADRC cohorts. The MoCA score ranges from one to 30, where only integer scores can be obtained. Participants with a total MOCA score greater than 24 were considered cognitively unimpaired (CU), and a MoCA score less than or equal to 24 was indicative of cognitive impairment (CI). A subset (91.1%) of 237 individuals in the EHBS cohort and 212 individuals in the ADRC cohort were screened for current depression symptomatology. The subset of subjects was not deliberately selected. Depression screening was part of the standard assessment for both cohorts; however, due to administrator error, patient fatigue, or other unforeseen problems, depression screening could not always be achieved. The EHBS and ADRC participants were recruited from parent studies which had slightly different protocol such as the choice of the different depression screening measures. As such, the depression measures used mirror the protocol for each study (EHBS used PHQ-8 and ADRC used BDI-II and GDS). The Beck Depression Inventory-II (BDI-II) [21] and the Geriatric Depression Scale (GDS) [22] were used to evaluate depression severity in the ADRC participants younger than 65 years of age and participants aged 65 years or older, respectively. Patients with a BDI-II score equal to or higher than 14 or with a GDS score higher than 5 were considered depressed. The Personal Health Questionnaire Depression Scale (PHQ-8) [23] was used to measure current depression in EHBS participants. Participants with a PHQ-8 score of 10 or greater were considered to have depression. Based on the corresponding scores, 40 participants from ADRC and one patient from EHBS were deemed to have current depression symptomatology.

Among the 235 ADRC participants, 232 (98.7%) of them have a clinical diagnosis record of a specific stage or etiology of cognitive impairment available and were included in the analysis of differentiating cognitive impairment across etiologies or disease stages. A subset of 74 (31.9% of the 232 ADRC participants with a record, with a MoCA of 12.9 ± 5.5) participants were diagnosed as AD-D, 33 (14.2%, MoCA: 26.6 ± 2.0) were diagnosed as subjective memory complaint (SMC), 61 (26.3%, MoCA: 20.2 ± 4.3) were diagnosed as AD-MCI, 19 (8.2%, MoCA: 21.3 ± 3.5) were diagnosed as non-AD-MCI (including vascular cognitive impairment, MCI from alcohol abuse and traumatic brain injury), and 45 (19.4%, MoCA: 16.5 ± 6.8) were diagnosed as non-AD-D (including DLB, frontotemporal lobar degeneration, vascular dementia, PD-D, alcoholic dementia and dementia from traumatic brain injury).

**Fig 1:**
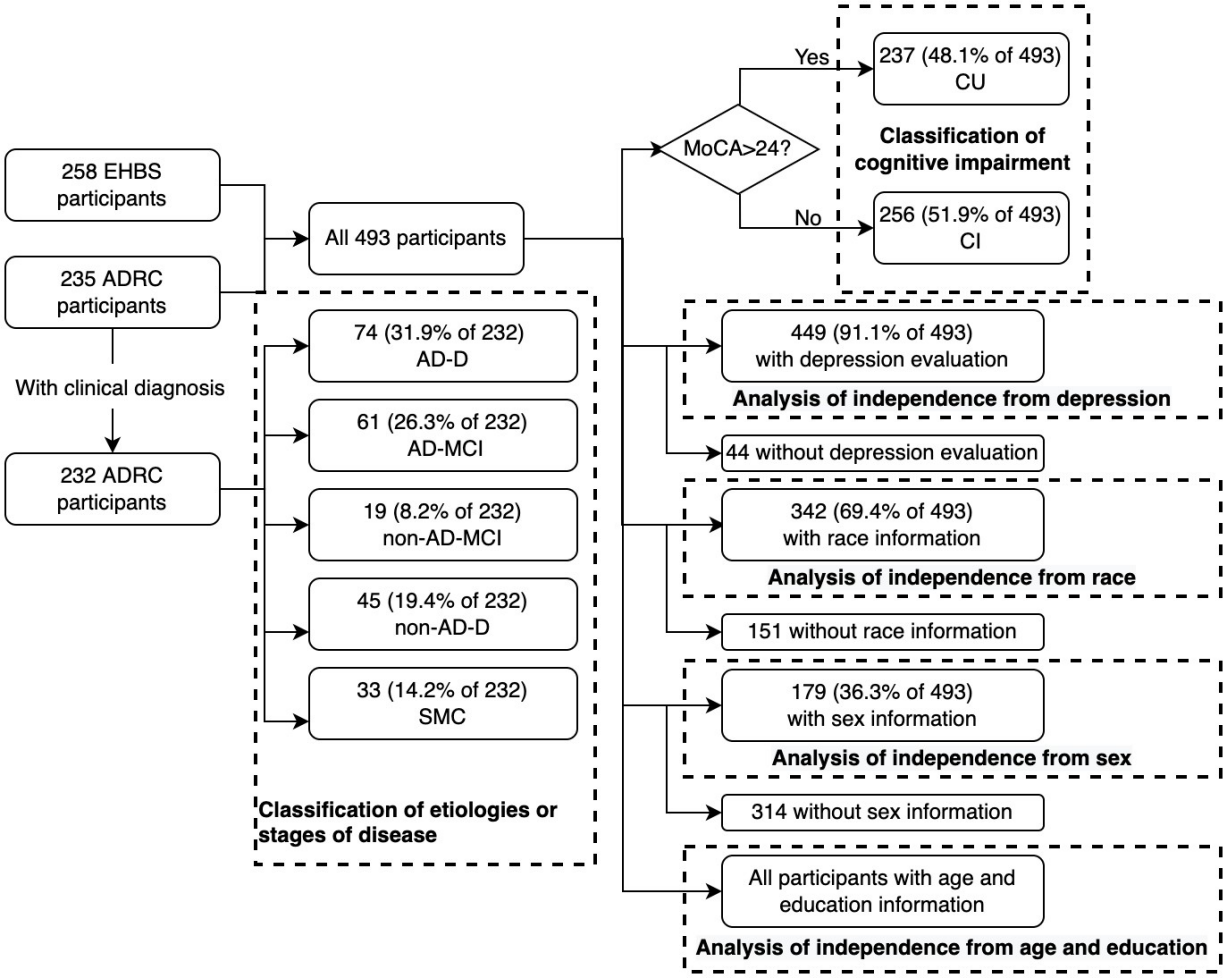
The flow of participants included in different analyses.

The subsets of participants included in different analyses can be found in the flow chart shown in Fig 1.

### Data collection

The details of the Visuospatial Memory Eyetracking Test was described in [24, 25], a passive viewing test that asks participants to enjoy the images displayed on the screen, where the participants were not asked to perform any memorizing task and did not get scores or any kind of feedback during the test. In short, the task first shows 20 images of scenes consisting of two to five objects for a duration of five seconds, then displays a modified set of images with either one object added or removed from each image. A total of 611 videos of the facial expressions during the memory test were collected from 493 subjects. Due to the presence of a separate, time-limited test-retest study which overlapped with the data collection of the current study, some participants had a second or third administration, which were deemed appropriate to be included in this study to provide additional information. In the second or third administrations, the participants did not undertake any cognitive tests (such as MoCA) before the proposed passive viewing test. Specifically, among the 493 participants, 385 participants took the test once, 98 participants took the test twice, and 10 participants took the test three times.

The memory test was administered to the participants using the same protocol described in [25]. Briefly, the test was presented on an iPad Air 9.7” tablet with maximum screen brightness and mounted on a stand in portrait orientation during the test. Each iPad was running at least iOS 10. The videos were captured from the tablet’s forward facing camera at a resolution of 720p and a sampling rate of 30Hz. The clinical testing rooms where the data were captured had both natural lighting from windows and overhead fluorescent or LED bulbs. During the calibration procedure, the participants were instructed to move their position to fit the silhouette of a face that appeared on the screen, resulting in an approximate distance of 350 mm between the iPad and the participant’s eyes.

### Vision-based eye tracking

We followed “Method 4” proposed in [25], in which we implemented a deep learning-based eye-tracking method to estimate the viewing time of the modified region in an image. The viewing time was defined as the percentage of frames (excluding the ones without face/eyes detected) where the estimated gaze is in a fixed expert-defined elliptical region (for each object/picture). The processing pipeline consists of: 1) a regression tree-based face and eye detection and cropping; 2) a convolutional neural network (CNN) for gaze location estimation trained on MIT’s GazeCapture dataset [26] on all the 611 recordings collected in this study; 3) a support vector regression (SVR) layer for gaze estimate calibration trained for each recording; and 4) a recalibraiotn of the SVR gaze estimation using a fixation cue between each image. The only modification in this new study presented here was the inclusion of a larger dataset based on the increased number of recordings (611 versus 250 recordings) in the Emory dataset. The average test error between the gaze estimate and the target in the test set was 1.98 cm on a 9.7” (24cm x 16.95cm) display.

### Vision-based facial expression recognition

To identify the facial emotion expressed during the test, we adopted the CNN based framework we proposed in [6]. For each frame of the recordings, the face of the participant is detected with Faster Regional-CNN [27] with a VGG16 [28] backbone network trained on the WIDER face dataset [29]. After segmentation, the face is fed into another CNN with VGG19 structure, which was trained on the AffectNet dataset [30], to estimate the probabilities of the facial emotion expressed being into seven categories, namely neutral, happiness, sadness, surprise, fear, disgust, and anger. This facial emotion classifier was tested on the evaluation set in the AffectNet dataset, in which subjects faces are captured ‘in the wild’. The emotion classifier was also evaluated on a subset of the Radboud Faces Database (RaFD) [31], where the participants are “front-facing”, or looking directly into the camera (as is the case for the experiments described here). The AffectNet database consists of 400,000 facial emotions in the wild settings and was collected from the web, while the RaFD was collected in a lab controlled environment.

#### Minimizing bias

We also tested the facial emotion classifier performance on images of people with different sexes, skin tones, and with or without occlusion. Since there is no available information provided in the original AffectNet dataset on these characteristics, the Microsoft Azure Face APIs were used to determine the sex, location of the outer tip of nose right alar, and whether the face was partially occluded. To estimate skin tone, on each image we sampled an area of 100 pixels (10 × 10) on the lower right of the outer tip of nose right alar. The average value of the red, green and blue channels were recorded separately. The resulting composite RGB representation was then matched to the closest one of the 36 colors in the von Luschan chromatic scale [32] and then converted to the closest one of the six Fitzpatrick skin types [33]. (The RGB representation for 36 colors in the von Luschan chromatic scale and the conversion rule can be found in S1 Table and S1 Fig).

The facial emotion classifier achieved an accuracy of 63.3% in the AffectNet evaluation set and 90.1% on the front-facing subset of the RaFD. In comparison, the accuracy of a random guess approach is 14.4%. Moreover, the agreement between two human annotators on the test set of AffectNet is only 60.7% [30].

We used 3446 images in the AffectNet evaluation set for performance assessment. Of these 51% of the images were deemed male and 49% were identified as likely to be female, 5% of the faces were partially occluded, and the distribution of the skin tones from type I to type VI were found to be 1.4%, 1.2%, 15.2%, 63.0%, 18.3%, 0.8%. The emotion classifier achieved an accuracy of 61.2% with emotions from male subjects, 65.6% accuracy with emotions from female subjects, 64.0% accuracy on all partially occluded subjects, and 63.3% accuracy on subjects without any partial face occlusion. Also, the classifier achieved an accuracy of 62.5%, 69.8%, 65.3%, 63.1%, 62.6%, 58.6% on skin types I through type VI (from lightest to darkest), respectively. There were no significant differences found in emotion classification accuracy between any pair of skin tones or between subjects with and without partially occluded faces. However, there we found a significant difference (McNemar’s test, *p* = 0.009) between the performance of the emotion classifier in males and in females (a 1.6% difference in the favor of males).

It is important to note that the AffectNet database has much lower quality than the RaFD database, with face orientation often away from the camera, with random lighting and scaling. The forward-facing images from the RaFD database maps more closely to the data we collected in the study presented here, in which our algorithm exceeded 90% average accuracy.

### Feature extraction

The average estimated probabilities of the facial emotions expressed during the test were used as the subject-level features to describe and classify the state of the subject. The average probabilities can also be viewed as “soft” frequencies of the emotions, in the sense that we averaged the probabilities instead of the presence of the dominant emotion in each frame. (i.e., there could be a face estimated to be half happy and half sad). Since the emotion classifier was trained on images of the presumed healthy and younger population from the AffectNet database, we hypothesized that the encoding from the facial characteristics to emotions in the population from this study might not be the same as that in the population represented in the AffectNet data, while the learned facial characteristics are generalizable across different populations. Hence, we also used as a feature the values of the penultimate layer output (PLO, i.e., the layer before the final emotion classification layer) of the emotion classification network for each frame. Intuitively the PLO might contain more generalizable facial representations that may be used for emotion classification, but contain less specific emotion encoding of the (AffectNet) population. Then we calculated the average of these penultimate layer outputs during the test.

In addition to the average probabilities of emotion or average penultimate layer output of the whole test, we also calculated the averages while viewing the original set of images and during viewing the modified set separately to test if the participants’ facial reactions to the modified images contain more information than the reference facial expressions expressed during viewing the original set.

For comparison, the average viewing times of the modified regions defined in [25] were also used as features.

### Classification of cognitive impairment

We conducted two classification analyses to test the following two hypotheses regarding the ability to classify cognitive impairment with reference facial expressions (during a passive test): (1) They could help differentiate Cognitively Impaired participants (CI) vs. Cognitively unimpaired participants (CU) and (2) they could help differentiate different etiologies or disease stages of cognitive impairments (including AD-dementia, AD-MCI, non-AD-dementia, non-AD-MCI and subjective memory complaint).

For the first analysis, we hypothesized that the reference facial expression is different in people with cognitive impairment compared to a healthy population. We approached this hypothesis by utilizing the facial expression features described above to classify each participant as cognitively impaired (CI, MoCA<= 24) or cognitive unimpaired (CU, MoCA> 24). All 493 ADRC and EHBS participants were included in this analysis. For those who took the test multiple times, each test was separately classified with the ground truth label (CI or CU) being the same across the multiple administrations. Demographic variables such as sex and education and other coexisting disorders such as depression may be associated with a higher probability of cognitive impairment [1]. We therefore examined the classification performances using age, sex, race, years of education and state of depression as features.

#### Decoupling from potential emotional influences from neuropsychological tests

As mentioned in Measurement, neuropsychological testings including MoCA were administered to all participants before the initial administration of the proposed memory test, which could potentially affect the emotions and facial expressions of the participants during the memory test. To investigate whether there is a significant effect in facial expressions from the neuropsychological tests, we compared the CU vs. CI classification performance (AUC) in the initial administrations with the performance in the second/third administrations. Because the participants did not undertake any neuropsychological test (such as MoCA) before the proposed memory test in the second or third administrations, if there is a significant effect caused by the neuropsychological tests, the differences in the second/third administrations should be smaller than the differences in the initial administrations. The classification performance in the second/third administrations should be lower as well, assuming the effect exists. To match the number of videos in the second/third administrations, a randomly selected subset of 118 videos in the initial administrations were used for the training and testing.

For the second analysis, we hypothesized that the reference facial expressions are different in people with cognitive impairment across different etiologies or stage of disease. This hypothesis is tested by using the features to classify the participants into five types of clinical diagnoses, including AD-MCI, non-AD-MCI, AD-D, non-AD-D and SMC. Only the 232 ADRC participants, who have a research diagnosis of a specific stage or etiology of cognitive impairment available, were included in this analysis. To provide a benchmark, we also tested whether the MoCA score can effectively differentiate these diagnoses.

Logistic regression (LR) with *l2* regularization and a support vector machine (SVM) with a radial-basis function kernel were used for the cognitive impairment classification. Multinomial LR and a one-vs-rest SVM were used for clinical diagnoses classification. These classifiers were implemented with Python Scikit-learn [34] package. We evaluated the cognitive impairment classification performance with two metrics: area under the receiver operating characteristic (AUC) and *F*_1_ score, which is the harmonic mean of the precision and recall. For the multi-class clinical diagnoses classification, we reported accuracy. All the metrics were calculated with five-fold cross-validation, where approximately 80% of the recordings were used for training, and approximately 20% were used for testing. Tests from the same participant were included in either training or testing set. This cross-validation was repeated 100 times where the five folds were randomly separated, and the averaged metrics were reported.

### Statistical analyses

Statistical tests were used to provide an assessment of the difference in the probability distributions of average emotions between CI and CU group and an assessment of performance from different features. The Shapiro-Wilk test was used to confirm that the distributions were not normally distributed. Thus, a two-sided Mann-Whitney rank test was applied between average emotions derived from subjects assessed as CI or CU to determine whether a significant difference exists between these two averages. The same tests between CI and CU were repeated on the subset of 408 subjects who were confirmed to have no current depression symptomatology since the state depression could potentially affect the distribution of facial expressions. McNemar’s test was used to evaluate the classification disagreement between pairs of classification settings. Chi-squared tests were used to determine whether significant differences were presented between the performance of the emotion classifier in images from different groups of people. Significance was assumed at a level of *p* < 0.05 for all tests.

## Results

### Comparing and combining memory and emotion metrics

#### Inter-group differences in emotional expression

**Fig 2:**
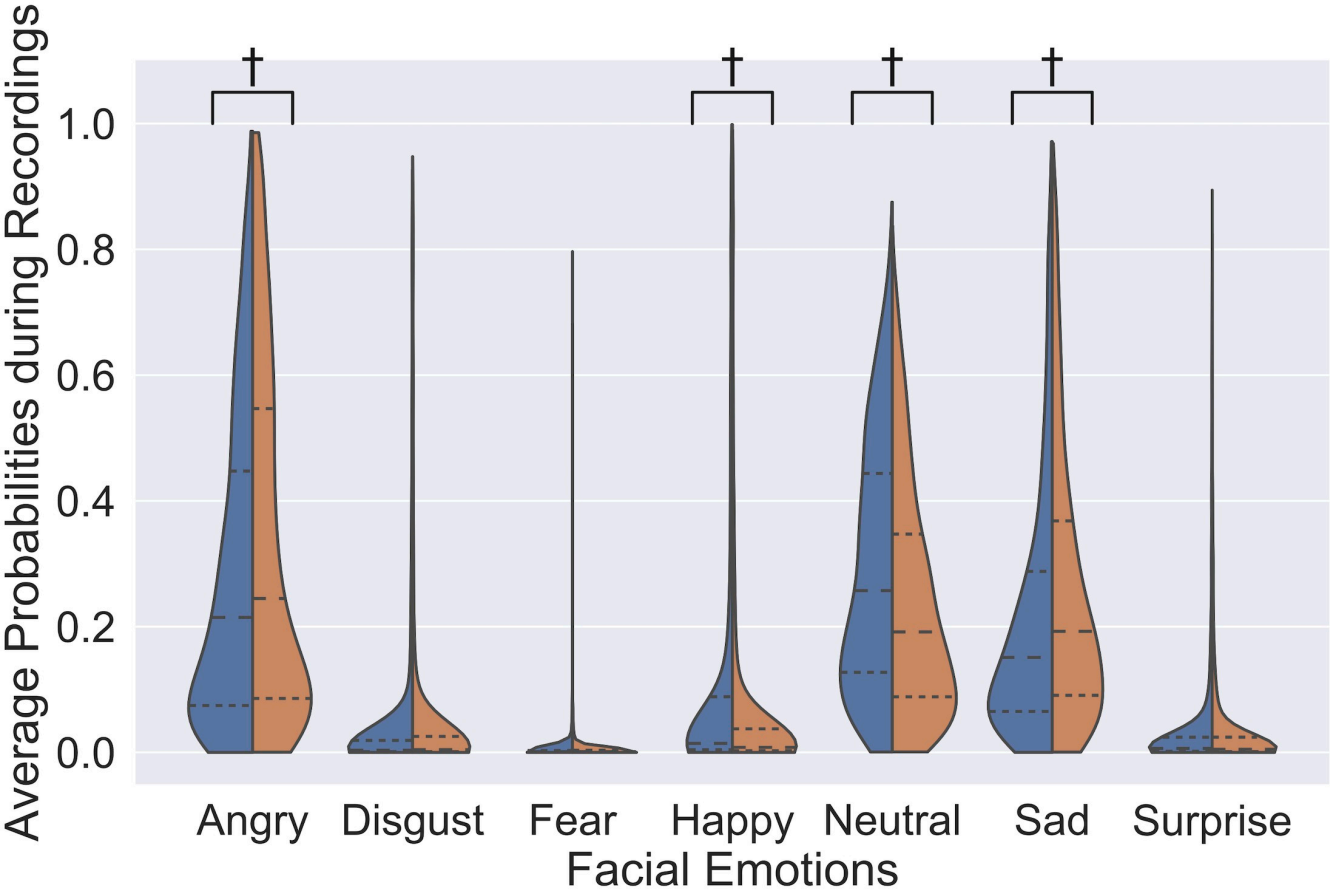
Distribution of average emotion probability in each recording in cognitively unimpaired participants (blue) and participants with cognitive impairment (orange). The inner quartile range and the average of the probability of each emotion within a certain group are depicted by dense and sparse dotted lines. Each distribution is smoothed using Gaussian kernel density estimation. † represents a significant difference in the median probability of emotion between groups at *p* < 0.05, assessed using a two-sided Mann-Whitney rank test.

Fig 2 illustrates the distribution of average probabilities of emotions during the test in the CU and CI groups. Two emotions, namely angry (*p* = 0.05), and sadness (*p* = 0.002) were found to be significantly less frequent in CU, while happiness (*p* = 8 × 10^−4^) and neural face (*p* = 4 × 10^−5^) were significantly more frequent in CU.

#### Cognitive impairment screening performance

Table 2 shows the performances in screening cognitive impairment using different types of features. Using the averages of the estimated probabilities of the facial emotions expressed during the test, the classifier achieved an AUC of 0.609 and an *F*_1_ score of 0.622.

**Table 2:** Classification performance of CU vs. CI.

Feature Type	Subjects	AUC	*F*_1_
1. Facial Emotions	493	0.609	0.622
2. PLO	493	0.657	0.620
3. Viewing Time	493	0.729	0.698
4. Age	493	0.669	0.648
5. Sex	179 (36.3%)	0.488	0.590
6. Race	342 (69.4%)	0.515	0.360
7. Education	493	0.642	0.633
8. Depression State	449 (91.1%)	0.552	0.248
9. Viewing Time+PLO ^†^	493	0.766	0.701
10. Age+PLO	493	0.677	0.636
11. Sex+PLO ^†^	179 (36.3%)	0.556	0.634
12. Race+PLO ^†^	342 (69.4%)	0.553	0.674
13. Education+PLO ^†^	493	0.698	0.642
14. Depression State+PLO ^†^	449 (91.1%)	0.654	0.606

Note: Depression state was coded as a binary variable for each participant, indicating whether the participant was depressed or not. The second column (Subjects) indicates the number of participants included in the classification. When the full cohort of 493 subjects could not be used due to missing information, a corresponding percentage of available subjects is provided in brackets. PLO indicates Penultimate Layer Output.

^†^ indicates that a statistically significant improvement was found when combining a type of feature with the penultimate layer features, compared to using that type of feature alone.

Logistic Regression was used as the classifier for all feature types except for facial emotions, where an SVM was used.

Additionally, using average penultimate layer output features improved the AUC to 0.659, indicating that more information was contained in them and empirically proving that our hypothesis about them being more suitable to be adopted for this population. Based on the coefficients of these learned classifiers, the top three important emotions for screening are neutral, sadness, and happiness.

The AUCs and *F*_1_ scores achieved through the use of these emotion-related features while viewing the original set of images and those while viewing the modified set were also compared, and no significant difference (McNemar’s test, *p* = 0.65) was found between the classification of these two classifiers. This result verified our assumption that the emotion-related features were mainly capturing the reference facial expression expressed during viewing the original set by the participants instead of the elicited ones.

In comparison, using the viewing time of the modified region resulted in an AUC of 0.729 and an *F*_1_ score of 0.698. When combining the viewing time and penultimate layer output (type 2 and 3), the performance improved significantly (McNemar’s test, *p* = 3), where the AUC was improved to 0.766 and the *F*_1_ score remained approximately unchanged.

The CU vs. CI classification performance (AUC) in the initial administrations was similar to the performance in the second/third administrations, which indicates that there was no significant effect on facial emotions expressed during the initial administration of the memory test caused by the neuropsychological tests administered before the memory test. To be more specific, no significant difference (McNemar’s test, *p* = 0.13) was found between the AUC (0.540) in the initial administrations and the AUC (0.526) in the second/third administrations. Please note that both performances are lower than the performances in all videos due to the significantly lower number (118, compared to 611) of available videos used in this decoupling analysis.

### Independence from demographics and other diagnoses

Based on these results, one question is whether the emotion estimation algorithm is simply capturing differences in patients due to factors such as age, sex, race, education, and other clinical issues such as depression. This section, therefore, presents an analysis of the contributions of these covariates to the classification.

#### Age, sex, race and education

From the fourth to the seventh row in Table 2 demonstrates the predictive power of the demographic variables, including age, sex, race, and years of education. Sex and race were found to be not very predictive, which was expected from Table 1, where sex was approximately equally distributed in the CI and CU groups, and Caucasian participants were the majority population in both groups. As shown in many previous studies, higher age and lower education were strongly associated with cognitive impairment, resulting in similar classification performances compared to facial expression-related features.

**Fig 3:**
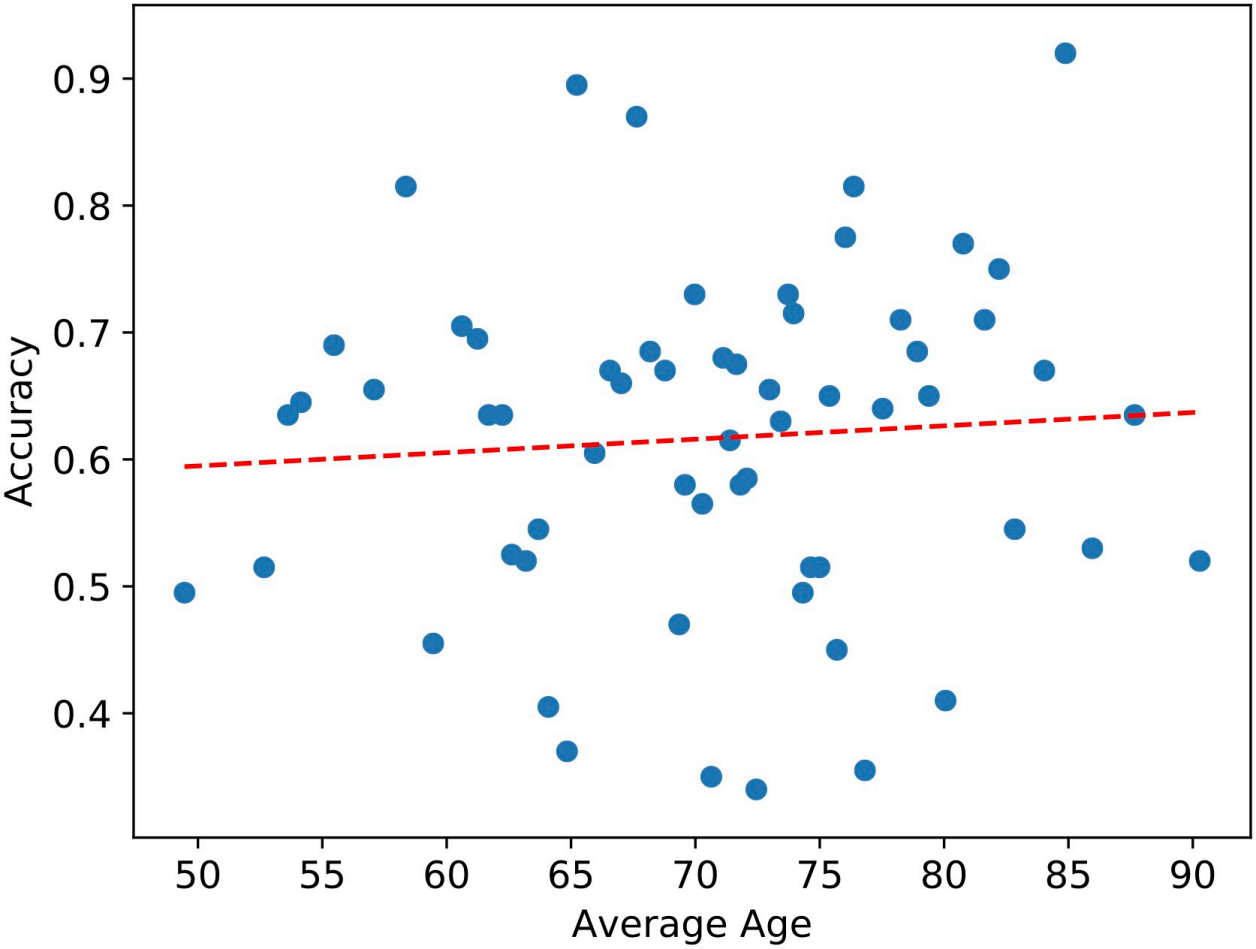
Accuracies of the classifiers using penultimate layer output as features at different ages. Each blue point represents ten participants, and the red dashed line is the linear trend line of the data.

Rows 10 to 13 in Table 1 show the performance using the combination of penultimate layer output features with each of the demographic features. The performance improved significantly (McNemar’s test, *p* < 0.001) when combining the penultimate layer output features with sex, race, or education. When combining with education, the AUC improved from 0.642 to 0.698, and the *F*_1_ score improved from 0.633 to 0.640. Although combining age with penultimate layer output features did not improve the AUC or the *F*_1_ score, we further investigated the relationship between age and the penultimate layer output features by plotting the accuracies of the classifiers using the penultimate layer output as features at different ages. (See Fig 3). No significant correlation was found visually or statistically (Spearman’s correlation test, *r*_*s*_ = 0.09, *p* = 0.48) between the accuracy and the age.

#### Depression

**Fig 4:**
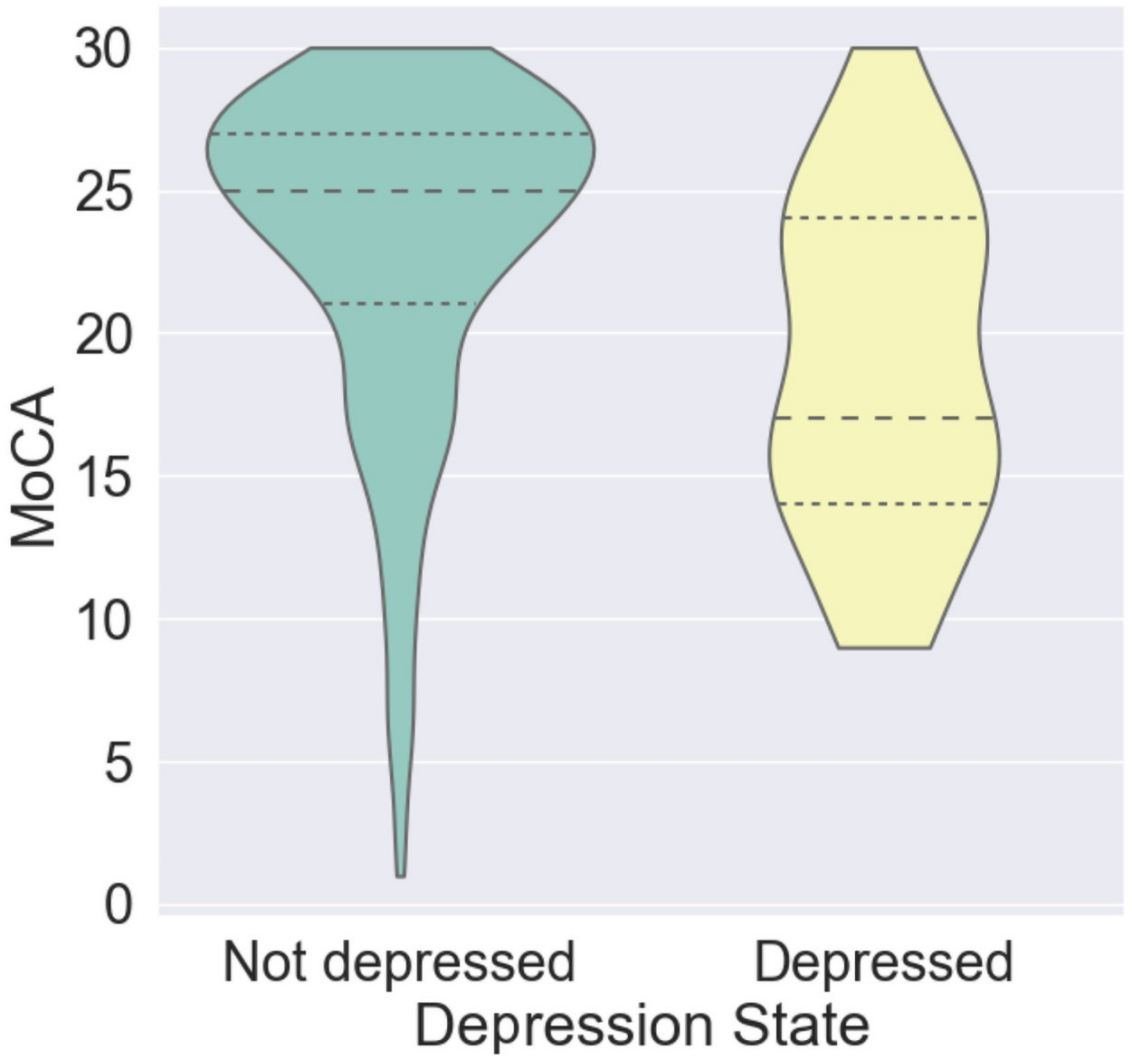
Distribution of MoCA scores in participants with depression (yellow) and without depression (blue). The inter quartile range and the average of the probability of each emotion within a given group are depicted by dense and sparse dotted lines. Each distribution is smoothed using Gaussian kernel density estimation.

Fig 4 illustrates the distribution of MoCA score in participants with depression and without depression. The states of depression of the participants in this analysis were decided by the results of the corresponding depression symptomatology screening described in the “Measurement” section.

As Fig 4 shows, there is significant overlap in the MoCA score between depressed and not depressed groups, resulting in the relatively poor predictive power of depression state for screening cognitive impairment. Similar evidence has also been showed in the eighth row of Table 2. Not surprisingly, utilizing the combination of penultimate layer output features and the depression state significantly (McNemar’s test, *p* < 0.001) improved the performance when compared to using the depression state alone. This indicates that facial expressions are not simply indirectly predicting the cognitive impairment state by capturing the depression state, which has been known to affect the facial expressions.

The results of the Mann-Whitney rank tests on the inter-group (CI vs. CU) differences in the emotional expression on the non-depressed participants agreed well with the results found on all participants described above in the ‘Inter-group differences in emotional expression’ section. To be more specific, among the participants who were confirmed to have no current depression symptomatology, angry (*p* = 0.05), and sadness (*p* = 0.01) were found to be significantly less frequent in CU, while happiness (*p* = 0.002) and neural face (*p* = 0.001) were significantly more frequent in CU.

### Differentiating subtypes of cognitive impairment

As shown in Table 3, neither facial expression related features (type 1 and 2) nor viewing time can be used to classify different kinds of cognitive impairment effectively. Nevertheless, information is present in these features since the accuracy is still higher than random guessing, which would provide an accuracy of 0.2. In comparison, using the MoCA score led to much better performance. However, using MoCA scores as the feature resulted in classifying all non-AD-D as AD-D, and all non-AD MCI as AD-MCI, because large overlaps in MoCA scores were presented within these two pairs respectively, and there are more AD-D and AD-MCI in the dataset.

**Table 3:** Classification performance of different diagnoses.

Feature Type	Classifier	Accuracy
1. Facial Emotions	SVM	0.276
2. Penultimate Layer Output	LR	0.257
3. Viewing Time Estimated by Eye-tracking	LR	0.275
4. MoCA score	LR	0.481

## Discussion

Results demonstrate that both eye-tracking (for measuring memory encoding) and quantification of emotional state via two different deep learning frameworks provided strong discrimination between cognitively impaired participants and cognitively unimpaired participants. Although viewing time provided better predictive power, it requires a more strict testing environment and more susceptible to noise, such as the change of holding angles. Combining both approaches provided a significant boost in performance, indicating that both memory and emotional expression are independently related to cognitive impairment at some level.

For the emotional expressions, we found that neutral, happiness, and sadness level contributed the most to the classification, which echoed the fact that there were significant differences in the expression of these three emotions between CI and CU. While hypomimia and slack facial expressions were found in types of non-AD dementia with known motor disturbances [17, 18], our quantitative analysis of a more diverse cognitively impaired group showed that neutral emotion was *less* expressed in cognitively impaired participants, indicating higher facial expressivity in CI.

The results showed that facial expressions in reaction to modified objects did not provide additional information for cognitive impairment classification, beyond the facial expressions while watching original images. Since many studies have found the elicited emotion to be useful for the classification, we argue that this result indicates that no significant expressions were stimulated by the modifications of the images, and only the overall emotional expressivity during the test was used to evaluate cognitive function. We have found that the CI displayed significantly more negative emotions (sadness and anger) during the tests, while the CU displayed significantly more positive emotion (happiness). This lack of expression of positive emotion in CI might help explain the discrepancy between our result and some previous studies [16] on facial expressivity in CI: because it is more difficult for a human to identify subtle negative emotions, based on the agreement rate in Mollahosseini *et al*. [30]. Therefore, a subtle increase in expression of negative emotions might be easily ignored by the observer, and the decreased expression of positive emotion may be more noticeable, leading to the previous impression of overall decreased expressivity. This hypothesis also helps explain the association between cognitive decline and increased facial expressiveness after controlling for apathy, as found in Seidl *et al*. [14]. Further investigation is needed to determine whether this tendency of reference emotional state of those individuals with cognitive impairment being more negative and less expressive is the direct consequence of their impairment.

In addition, the results found were largely independent of race, sex, education level, and the existence of depression since combining facial emotional features (Penultimate Layer Output) with each of them significantly improved the performance in cognitive impairment screening. Due to the missing information of race, sex and existence of depression on some subjects, the independence of facial expressions from those partially available variables is limited to the subset of subjects with those variables available. However, the statistically significant (*p* < 0.001 for all three variables) improvements in performance suggest that this difference is highly likely to be generalizable in all participants. Combining facial emotional features with age did not improve the performance compared to using age alone, which indicates that age may contribute simultaneously to the decline of cognitive function and the changes in facial emotion expressions. Nevertheless, no significant correlation was found visually or statistically between the classifier performance with facial emotional features and the age, showing that classifications made with facial emotional features were independent of age.

Cognitive impairment screening results showed that using penultimate layer output features boosted performance, indicating that the facial characteristics themselves contain more information than the encoding from the facial characteristics to emotions. This result could be explained by the fact that the emotion classifier was trained on (presumed) healthy population and was applied to the cognitively impaired population. However, training the emotion classifier directly in the cognitively impaired population is also problematic. Firstly, it is time and financially expensive to collect and label a large number of facial expressions in this population. Secondly, the labels of the facial expression can be even noisier and difficult to be determined in cognitively impaired population because their external expression and internal feelings can be very different. Hence, machine learning methods such as domain adaption could be used in the future to help learn the representation of the facial characteristics that are both effective in healthy controls and cognitively impaired populations.

Although the performance was improved by using the penultimate layer output features, the interpretability of the classifier was much lower than directly using the facial emotions, where we can clearly state which emotions played the most important roles in screening cognitive impairment. One possible approach to mitigate this lack of interpretability is to visualize the regions on each participant’s face that contribute the most to the classification of the participant as cognitively impaired. For example, Gradient-weighted Class Activation Mapping [35] can be used to produce a coarse localization map highlighting such regions.

A potential limitation of this study is that the differences of the reference facial expression during the memory test might be caused by the potential stronger stress experienced during other cognitive tests (such as MoCA) by CI participants when compared to CU participants. However, the following evidence suggests that this is not the main reason for the observed differences. First of all, we have demonstrated in the experiments that the CU vs. CI classification performance (AUC) in the initial administrations is similar to the performance in the second/third administrations. The similar predictive powers indicate that the observed differences were at a similar level in the initial administration, which provides strong evidence that they were not caused by the cognitive tests administered before the memory test. In addition, if the potential stronger stress from previous cognitive tests for CI participants (compared to CU participants) are causing the differences in facial expressions, its effect is likely to decrease over time during the memory test since the test was designed to be relaxing, but no significant changes of facial expressions over time were found for either CU or CI. Although decreased expression of positive emotion and increased expression of negative emotions could potentially be explained by stress, this may be underlying stress. Moreover, it is nontrivial to attribute the increased facial expressiveness in CI subjects to stress and could be due to other changes in disease-related brain processing or the ability to control facial expression.

Another limitation of this study is that the reference emotion expression during the lab-controlled environment may not necessarily be the same as those expressed during normal activities. Though the results indicated that the test did not stimulate any significant emotional changes, further investigation is needed to be able to extend our findings of reference emotion expressions to facial emotion expression patterns during daily activities. For instance, edge computing devices could be used to capture patients’ emotional expressions during activities at home or in the hospital.

It is important to note that the approach we described in this article is unbiased with respect to skin tone. However, there was a small performance drop (of% 1.6 overall) in the emotion classifier when applied to females versus males. There is a possibility that this difference is due to the slight difference in proportion of male and female emotions (51% vs. 49% respectively) in the training examples used in the original AffectNet work. However, the large size of the AffectNet data and the small difference in proportions makes this unlikely. The performance drop may be primarily be due to the known differences of emotional expression in different sexes [36] and the resulting difference in the ability to recognize emotions expressed by people of different sex [37]. Notably, sex differences in emotional expression have been observed in patients with cognitive impairment [38]. Therefore, due to those differences, the cognitive impairment classifier with emotion features was found to perform differently (Chi-squared test, p = 0.01) in different sexes (n = 179), achieving an accuracy of 59.1% in females and an accuracy of 72.4% in males. Further investigations on the sex differences of emotional expression and a larger number of subjects are needed to determine the validity of the explanations, and causes of the resulting performance bias.

It is also important to note that inter-nation [39] and intra-nation [40] cultural differences in emotional expression and processing have been reported in previous studies. Such differences could lead to cultural bias in the emotion classifier due to the biased distribution of training emotions and biased annotation of emotions. We were unable to quantitatively test differences in the performance of our classifier due to cultural differences. Qualitatively speaking, the training data (AffectNet) that we used has a strong potential to be culturally diverse because it was drawn from online images searched explicitly with different ethnicity and searched in six different languages [30]. However, the cultural diversity in annotation might be limited since all the emotion annotations were made by annotators at the University of Denver. Moreover, known biases in populations who have access to technology and the internet [41] are likely to bias the available data to some extent.

Lastly, the relative ineffectiveness in differentiating subtypes of cognitive impairment using emotions, eye tracking, or MoCA test suggests that increased numbers of individuals with defined non-AD etiologies are needed to identify the subtle differences in the various cognitiviely impaired subtypes.

## Conclusion

In conclusion, a deep learning-based analysis of facial emotion expression in 493 healthy controls and patients with various types of cognitive impairments provided evidence that the reference facial emotions red expressed in a lab-controlled environment are significantly different in people with cognitive impairment. More specifically, decreased expression of positive emotion, increased expression of negative emotions, and increased facial expressiveness were detected in a cognitively impaired population, compared to controls. In addition, these differences in emotional expression can be used to effectively screen for cognitive impairment. We note that the analysis is largely independent of age, race, sex, education level, and the existence of depression. Lastly, the combination of facial expression analysis and eye-tracking was shown to significantly improve the effectiveness of the approach described in this work for cognitive impairment screening, when compared to using eye-tracking alone.

## Supporting information

S1 Fig A reproduction of the von Luschan’s chromatic scale made by anthropologist Felix von Luschan. It was adapted from Felix von Luschan Skin Color chart on Wikimedia Commons (available from https://commons.wikimedia.org/w/index.php?title=File:Felix_von_Luschan_Skin_Color_chart.svg&oldid=473267354) under the Creative Commons CC BY SA license.
(TIF)

S2 Fig Distribution of average emotion probability in each recording in cognitively unimpaired participants (blue) and participants with cognitive impairment (orange) on the non-depressed participants. The inter-quartile range and the average of the probability of each emotion within a certain group are depicted by dense and sparse dotted lines respectively. Each distribution is smoothed using Gaussian kernel density estimation. † represents a significant difference in the median probability of emotion between groups at *p* < 0.05, assessed using a two-sided Mann-Whitney rank test.
(TIF)

S1 Table RGB representation of the skin types. vLST: von Luschan Skin Type [32], FST: Fitzpatrick Skin Type [33], RGB: values in red, green, blue channel.
(TIF)
