# Supplementary figures and images for: Automated analysis of facial emotions in subjects with cognitive impairment
Source: PLoS One. 2022 Jan 21;17(1):e0262527. doi: 10.1371/journal.pone.0262527 (PMC8782312; doi:10.1371/journal.pone.0262527)

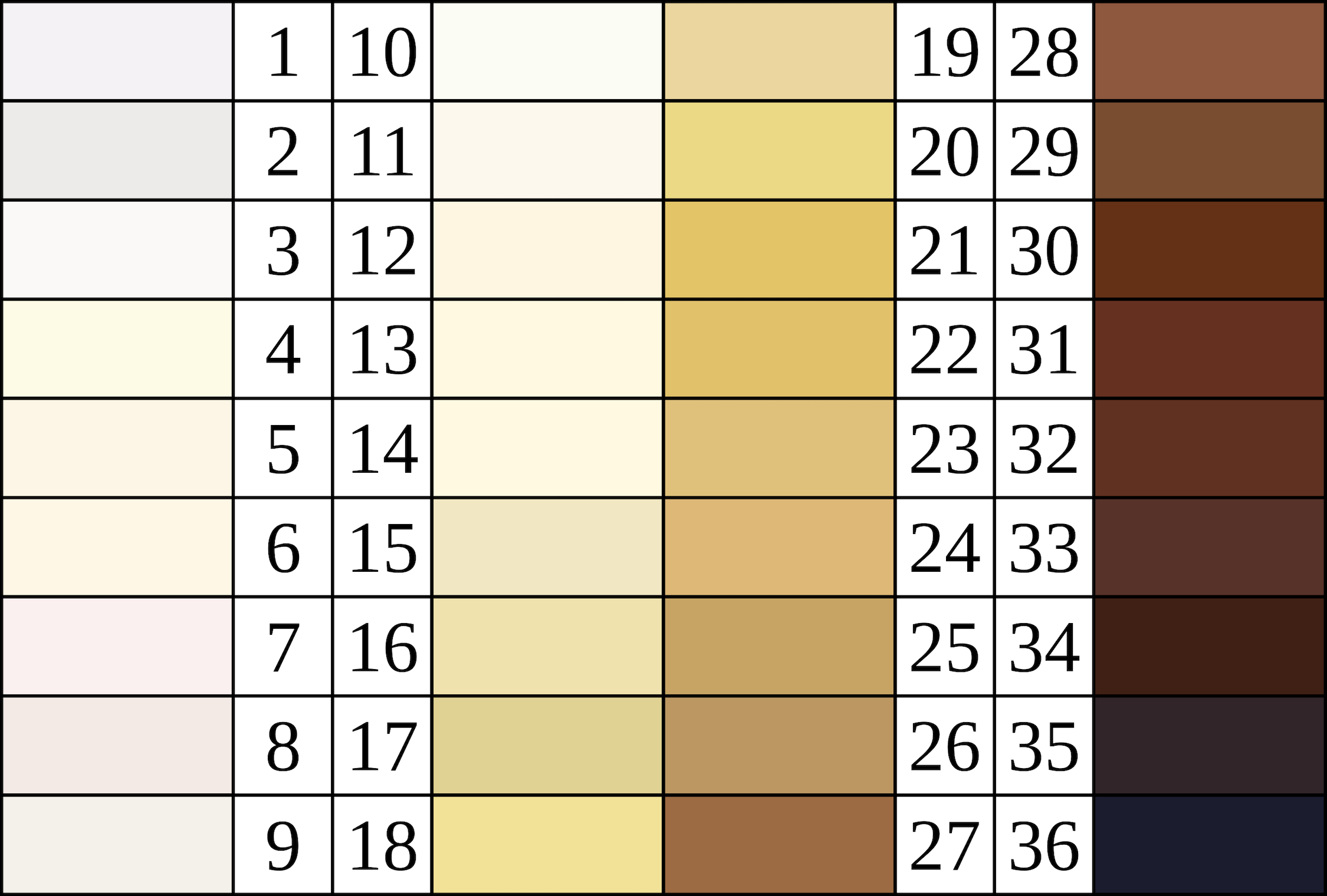

Supplement: S1 Fig — It was adapted from Felix von Luschan Skin Color chart on Wikimedia Commons (available from https://commons.wikimedia.org/w/index.php?title=File:Felix_von_Luschan_Skin_Color_chart.svg&oldid=473267354) under the Creative Commons CC BY SA license. (TIF) [file pone.0262527.s001.tif]

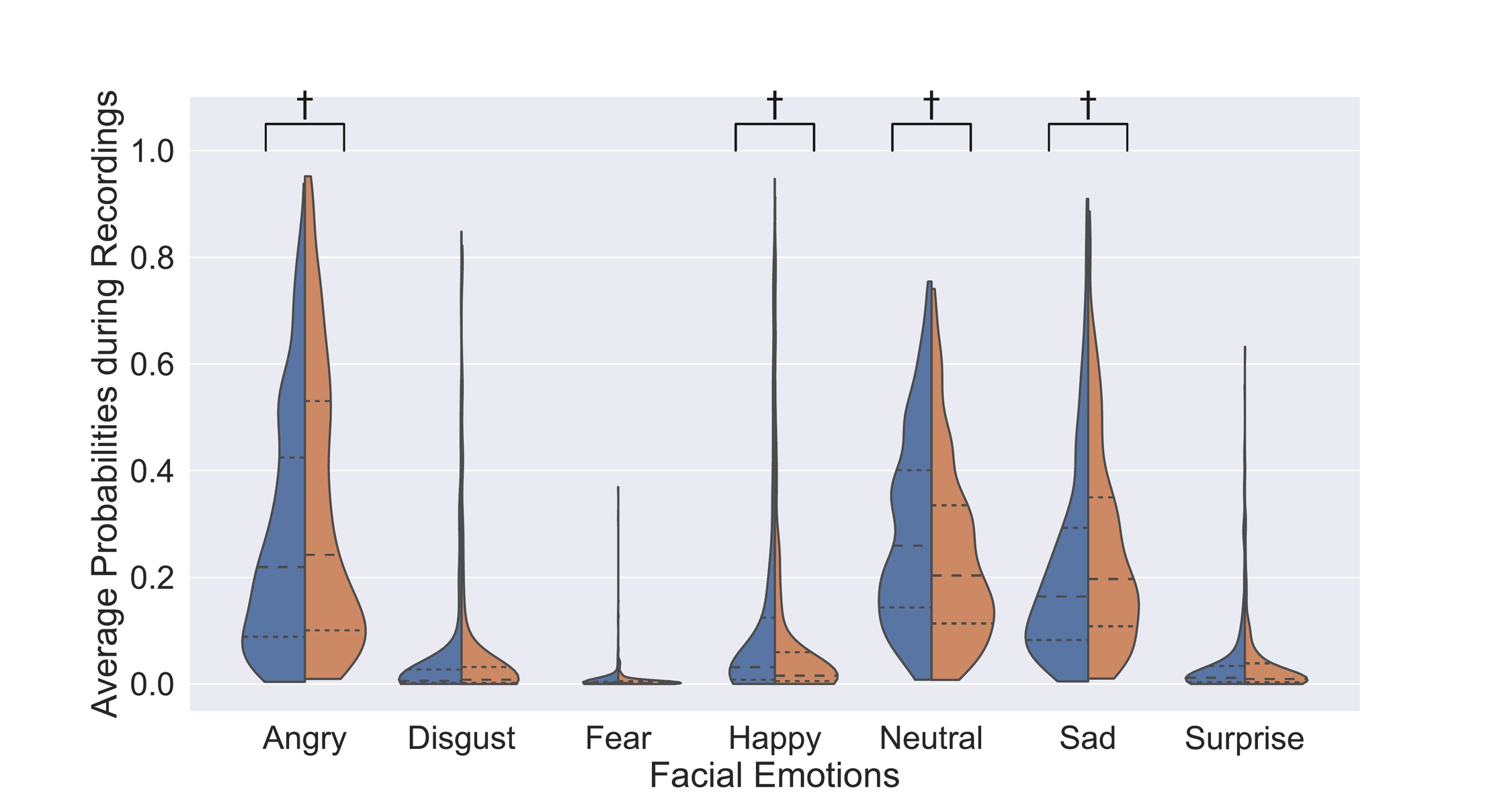

Supplement: S2 Fig — The inter-quartile range and the average of the probability of each emotion within a certain group are depicted by dense and sparse dotted lines respectively. Each distribution is smoothed using Gaussian kernel density estimation. † represents a significant difference in the median probability of emotion between groups at p < 0.05, assessed using a two-sided Mann-Whitney rank test. (TIF) [file pone.0262527.s002.tif]

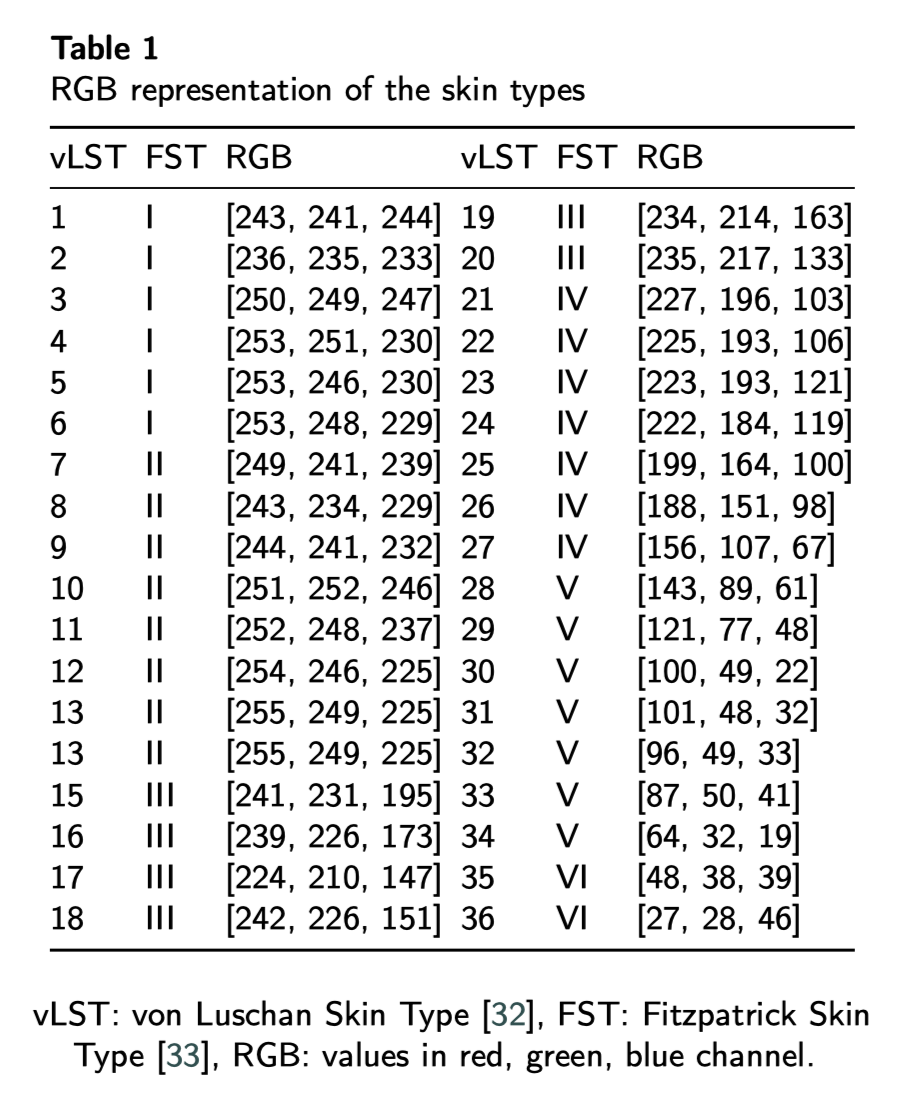

Supplement: S1 Table — vLST: von Luschan Skin Type [32], FST: Fitzpatrick Skin Type [33], RGB: values in red, green, blue channel. (TIF) [file pone.0262527.s003.tif]
